# A prospective survey of critical care procedures performed by physicians in helicopter emergency medical service: is clinical exposure enough to stay proficient?

**DOI:** 10.1186/s13049-015-0128-9

**Published:** 2015-06-11

**Authors:** Stephen J. M. Sollid, Per P. Bredmose, Anders R. Nakstad, Mårten Sandberg

**Affiliations:** Air Ambulance Department, Oslo University Hospital, Postboks 4956 Nydalen, 0424 Oslo, Norway; Faculty of Social Sciences, University of Stavanger, Stavanger, Norway; Norwegian Air Ambulance Foundation, Drøbak, Norway; Faculty of Medicine, University of Oslo, Oslo, Norway

**Keywords:** Helicopter emergency medical service, Emergency medicine, Emergency critical care, Prehospital procedures

## Abstract

**Background:**

Physicians in prehospital care must be proficient in critical care procedures. Procedure proficiency requires a combination of training, experience and continuous clinical exposure. Most physicians in helicopter emergency medical service (HEMS) in Norway are well-trained and experienced anaesthesiologists, but we know little about their exposure to critical care procedures in the prehospital arena. This knowledge is required to plan clinical training and in-hospital practice to maintain core competences for a HEMS physician.

**Methods:**

We collected survey data on critical care procedures performed by physicians at three HEMS bases in Norway for a one-year period. To correct for differences in duty time between physicians, the expected number of procedures performed in a full time engagement at each HEMS base was calculated. Data was analysed using descriptive statistics and expected procedure volume at each base was compared using one-way between group analysis of variance.

**Results:**

We received data from 82.7 % of the duty hours in the study period. Physicians at Oslo University Hospital HEMS had the highest volume of procedures in most categories and were expected to perform a majority of the procedures at least once a year. There were significant differences in procedure volume between the bases in 25 procedures.

**Conclusions:**

Physicians in Norwegian HEMS perform critical care procedures with variable frequencies. The low procedure volume in some cases and variance between bases indicate the need for a tailored procedure maintenance training and relevant in-hospital clinical practice.

**Electronic supplementary material:**

The online version of this article (doi:10.1186/s13049-015-0128-9) contains supplementary material, which is available to authorized users.

## Background

In prehospital critical care a core set of skills are required to have the ability of performing time critical procedures. Some of these procedures, e.g. endotracheal intubation (ETI), controlled ventilation and chest tube drainage, have been shown to contribute to improve patient outcome when performed prehospital by specially trained physicians [1]. Lifesaving skills and procedures are however often performed irregularly in the prehospital arena and in changing and challenging environments with little or no previous planning. It is therefore important to be sufficiently proficient in these skills.

Previous studies have indicated that for some skills the provider must perform a certain number of procedures before he/she is regarded as proficient [2, 3]. Studies on advanced life support (ALS) show that skills decay with time, and that retraining is necessary to maintain skills [4]. Clinical experience seems to slow the decay of skills proficiency, but how much clinical experience is necessary or how often skills must be retrained remains unknown [4].

In the Norwegian Emergency Medical Service (EMS) system, the anaesthesiologists are part of a two-tired response system using rapid response cars or helicopters [5]. The majority of the anaesthesiologists also work in-hospital in anaesthesia and critical care to ensure that core competences are maintained. However, there is no consensus to the amount or type of in-hospital clinical practice that is needed. A recently established standard for physicians in helicopter EMS (HEMS) in Norway states that a variety of clinical areas should be covered during in-hospital practice, including cardiology, paediatrics, obstetrics, neurology and traumatology [6]. However, this standard is based on the opinions of a working-group that developed the standard and not on evidence.

We believe that the individual volume of certain critical procedures performed prehospital by EMS physicians is relatively low compared to similar procedures performed by specialized physicians in-hospital. However, we do not know how big these differences are. Consequently we cannot determine what kind of in-hospital clinical practice is best suited for EMS physicians to maintain their procedure proficiency. Data on performed procedures can usually be found in activity data records from the EMS services, but in many cases these data only state what was done and not who actually did it. To increase our knowledge of the volume of critical procedures that are performed by EMS physicians, as a basis for further discussions on the need for clinical practice and training, we prospectively recorded all procedures performed by the physicians at two HEMS bases and one Search and Rescue (SAR) base in Norway during 12 months. Our hypothesis was that the volume of certain critical skills and procedures performed by individual physicians is very low.

## Methods

### Study population

There are 11 civilian HEMS bases in Norway. Ten HEMS units are staffed by a crew consisting of a pilot, a HEMS rescue paramedic and an anaesthesiologist. One base also includes an anaesthetic nurse in the crew. The Norwegian HEMS is used for primary missions in both trauma and non-trauma cases of all ages, as well as for transfer of critically ill patients between hospitals. All bases are operational day and night throughout the year. In addition to the civilian HEMS, six SAR helicopters are used for ambulance missions and are staffed with an anaesthesiologist in addition to the military crew.

The HEMS base of Stavanger University Hospital (SUH) covers a mixed urban and rural population of approximately 500.000 people with one helicopter. The helicopter at SUH HEMS carried out 754 missions in 2011. At Oslo University Hospital (OUH), two helicopters cover a mixed urban and rural population of approximately 2 million people. A separately staffed physician-staffed ambulance not part of this study also covers the city area of Oslo with approximately 600,000 inhabitants. The two helicopters at OUH HEMS carried out 895 and 651 missions, respectively, in 2011. The SAR helicopter at Rygge (Rygge SAR) is located in the catchment area of the HEMS of OUH and acts as a supplement to the civilian HEMS. Rygge SAR carried out 167 missions in 2011; 117 were SAR missions and 50 were either primary or interhospital transfer missions. All HEMS and SAR bases are equipped with rapid response vehicles for missions in the close vicinity of the base or for missions where, for any reason, helicopters cannot be used. These missions are also included in this study.

The physicians in the HEMS services of both SUH and OUH have rotations that include in-hospital duty in anaesthesiology. At SUH the physicians are employed in the Department of anaesthesiology and intensive care and sectioned for HEMS duty for different time periods. Some of the physicians in the department that have previously been sectioned for HEMS duty also do single HEMS watches as extra duty. At OUH all physicians are employed in the Air Ambulance Department and rotate for one week of in-hospital duty in anaesthesiology in one of four different hospitals every six weeks. At both SUH and OUH some of the physicians have reduced overall clinical working hours due to research activity. The physicians working at Rygge SAR do not have a fixed rotation, but are obliged to serve at Rygge SAR between four and six weeks every year on top of their regular rotations at their respective hospital and anaesthesiology department.

### Data collection

Throughout 2011 all physicians serving at SUH HEMS, OUH HEMS and Rygge SAR were asked to document the number of procedures they had performed during one shift or a maximum of 24 h. The normal shift length at SUH HEMS is 24 h, at OUH HEMS between 48 or 72 h and at Rygge SAR between 3 and 7 days. Some of the procedures recorded are “team-skills”, e.g. advanced cardiac life support (ACLS) or ETI. We therefore specifically asked the physicians to record procedures were they had been the protagonists of the procedure, i.e. they were “hands-on” during ACLS or leading the resuscitation, or they intubated the patient themselves with assistance from others. The procedures were recorded manually on a paper-based form with a predefined list of procedures at the end of each shift or after 24 h (Additional file 1: Table S1). The list was composed by the authors based on their experience as physicians in HEMS. All data were manually typed into a FileMaker database (FileMaker Inc, Santa Clara, CA, USA) by one of the authors (SJMS).

### Data analysis

**Fig. 1:**
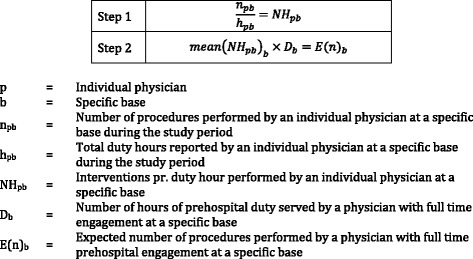
Method for calculating expected number of procedures performed prehospital in one year by an average physician with full time engagement

We used descriptive statistics to describe the data collected. This analysis included percentages, minimum and maximum values and mean values with Interquartile Ranges (ICR). Because there were huge variances in duty hours between the physicians involved in the study, we correct for the differences in duty hours by calculating the expected number of procedures that would be performed prehospital in one year by a physician with full time engagement at each base. This provided us with a generic procedure volume for the physicians at each base. The method for calculating the value of expected number of procedures is shown in Fig. 1. For the OUH and SUH HEMS, a full time engagement involves one week of prehospital duty in a six-week rotation, which equals 61 24-h shifts in a year. Physicians at Rygge SAR have a total of six weeks of duty in one year, which corresponds to 42 24-h shifts.

To compare for differences in procedure volume between the bases we also calculated the number of procedures performed per reported hour (n/x hours) and compared the mean values using One-way between-groups analysis of variance (ANOVA). A *p*-value < 0.05 was considered statistically significant.

Statistics were calculated using SPSS Statistics 22 for Macintosh (IBM Corp., Armonk, NY, USA).

### Ethics

The Data Protection Official at OUH and SUH approved the study as a quality improvement project and stated that formal approval from the Regional Committee for Medical and Health Research Ethics was not necessary.

## Results

We received data from 82.7 % of the 24-h periods covered by the study (96.6 % from OUH HEMS Helicopter 1, 96.2 % from OUH HEMS Helicopter 2, 69.3 % from SUH HEMS and 68.6 % from Rygge SAR). Thirty-six anaesthesiologists reported their activities during the study period. Six physicians were active at three of the helicopters in the study (OUH HEMS Helicopter 1 and 2 and Rygge SAR), and eleven were active at two helicopters (OUH HEMS Helicopter 1 and 2). The number of hours of prehospital duty that each physician reported ranged from 24 to 2326 h (1–97 24 h shifts), with a mean value of 783.1 h (IQR 276, 1287).

Table 1 lists all procedures that a physician with a full time engagement at one of the bases is expected to perform at least once a year; at OUH HEMS this was the case in 30 procedures, at SUH HEMS in 24 procedures and at Rygge SAR in only seven procedures. As an example of less often performed procedures, Table 2 lists procedures that a physician with full time engagement at OUH HEMS is expected to performed less frequently than once a year. Among the procedures listed in Table 2 are critical procedures like cricothyrotomy, assistance to child-birth and ETI in children.

**Table 1:** Procedures expected to be performed by a prehospital physician at least once a year. The average number of procedures is based on 61 24-h shifts (All, OUH HEMS and SUH HEMS) or 42 24 h shifts (Rygge SAR) per year. Numbers apply for all age groups if nothing else is noted

		OUH HEMS	SUH HEMS	Rygge SAR
BMV in patient	1–12 yoa	0.4	1.5	0.2
	>12 yoa	7.5	26.6	1.8
ETI in patient	<1 yoa, all categories	1.1	0.0	0.2
	1–12 yoa, all categories	1.3	1.0	0.2
	>12 yoa, other	5.2	6.3	0.0
	>12 yoa, respiratory distress	1.3	1.2	0.0
	>12 yoa, cardiac arrest	7.8	16.3	2.5
	>12 yoa, trauma	5.0	3.9	0.8
Inhalation therapy		1.4	1.0	0.3
Invasive ventilator in patient	<1 yoa	2.0	0.0	0.0
	1–12 yoa	2.3	0.0	0.0
	>12 yoa	36.1	9.3	1.3
Non-invasive ventilator in patient >12 yoa		2.7	1.0	0.3
ACLS in patient >12 yoa		13.8	23.4	3.2
Anti arrhythmic therapy		2.4	1.0	0.3
Vasopressor initiated		5.6	1.7	0.5
Vasopressor continued		12.0	3.2	0.0
Peripheral venous access in patient	<1 yoa	1.4	0.2	0.3
	1–12 yoa	3.9	2.7	0.3
	>12 yoa	30.4	44.1	5.1
Intra-osseous access in patient >12 yoa		1.6	3.9	0.3
Central venous catheter insertion		3.6	0.2	0.0
Arterial line insertion		8.8	1.2	0.5
Incubator transport	without ventilator	1.7	0.2	0.0
	with CPAP	2.2	0.0	0.0
	with ventilator support	2.9	0.0	0.0
Anaesthesia induction		25.3	11.0	1.5
Fracture repositioned		2.0	3.7	0.3
Dislocated joint repositioned		0.1	1.2	0.0
Pain management		8.8	9.3	1.7
Chest drainage		1.7	1.2	0.0
Gastric tube insertion		4.3	4.1	0.3

*ACLS* Advanced Cardiac Life Support, *BMV* Bag Mask Ventilation, *CPAP* Continuous Positive Airway Pressure, *ETI* Endotracheal Intubation, *OUH HEMS* Oslo University Hospital Helicopter Emergency Service, *Rygge SAR* Search and Rescue helicopter at Rygge, *SUH HEMS* Stavanger University Hospital Helicopter Emergency Service, *yoa* years of age

**Table 2:** Procedures performed less frequent than once a year. Procedures that are expected to be performed less frequent than once a year by a prehospital physician at OUH HEMS. Occurrence is based on 61 24-h shifts per year. Numbers apply for all age groups if nothing else is noted

Procedures expected to be performed once between every 1 to 2 years
• BMV in patient < 1 yoa
• Endotracheal intubation in patient <1 yoa with cardiac arrest
• Endotracheal intubation in patient 1–12 yoa with cardiac arrest
• Non-invasive ventilator in patient < 1 yoa
• Intra-osseous access in patient 1–12 yoa
Procedures expected to be performed once between every 2 to 5 years
• BMV in patient 1–12 yoa
• Endotracheal intubation in patient <1 yoa, other causes
• Supraglottic airway device in patient >12 yoa
• Non-invasive ventilator in patient 1–12 yoa
• Advanced cardiac life support in patient <1 yoa
• External cardiac pacing
• Intra-osseous access in patient <1 yoa
• Needle chest decompression
• Local anaesthesia
Procedures expected to be performed with more than 5 year intervals
• Endotracheal intubation in patient <1 yoa with respiratory distress
• Endotracheal intubation in patient <1 yoa with trauma
• Endotracheal intubation in patient 1–12 yoa, other causes
• Endotracheal intubation in patient 1–12 yoa, with respiratory distress
• Endotracheal intubation in patient 1–12 yoa with trauma
• Supraglottic airway device in patient <1 yoa
• Supraglottic airway device in patient 1–12 yoa
• Cricothyrotomy
• Advanced cardiac life support in patient 1–12 yoa
• Umbilical cord catheter insertion
• Birth assistance
• Reposition of dislocated joint
• Urethral catheter insertion

*BMV* Bag mask ventilation, *CPAP* Continuous Positive Airway Pressure, *OUH HEMS* Oslo University Hospital Helicopter Emergency Medical Service, *yoa* years of age

OUH HEMS had the highest frequency of procedures performed in most categories (36 of 58), but we found significant differences in procedure volume between the bases in only 25 procedures (Table 3). Some of these differences were attributed to the fact than one of the bases had not carried out the procedure at all. OUH HEMS had a significantly higher number of procedures in 11 categories; invasive ventilator treatment in children, central venous catheter and arterial line insertion, incubator transports, vasopressor treatment and anaesthesia induction. SUH HEMS had a significantly higher frequency of procedures in seven categories; ETI in adults in general and with cardiac arrest, bag mask ventilation (BMV) in adults, peripheral venous catheter and intraosseus needle insertion, ACLS in adults and repositioning of dislocated joints.

**Table 3:** Procedures with significant difference in mean value of procedures pr. hour between two bases. The base with the highest value is listed in the left column

Variable	Base	Mean	95 % CI	Base	Mean	95 % CI	*p* value
ETI >12 yoa, trauma	OUH HEMS	0.0031	0.0022, 0.0040	Rygge SAR	0.0008	0.0001, 0.0015	0.017
ETI >12 yoa, cardiac arrest	SUH HEMS	0.0117	0.0089, 0.0146	OUH HEMS	0.0050	0.0039, 0.0061	0.000
	SUH HEMS	0.0117	0.0089, 0.0146	Rygge SAR	0.0025	0.0012, 0.0037	0.000
ETI >12 yoa, other	OUH HEMS	0.0034	0.0024, 0.0044	Rygge SAR	0.0000	0.0000, 0.0000	0.001
	SUH HEMS	0.0052	0.0031, 0.0073	Rygge SAR	0.0000	0.0000, 0.0000	0.000
ETI >12 yoa, all categories	OUH HEMS	0.0034	0.0101, 0.0135	Rygge SAR	0.0000	0.0018, 0.0047	0.000
	SUH HEMS	0.0052	0.0149, 0.0227	OUH HEMS	0.0034	0.0101, 0.0135	0.000
	SUH HEMS	0.0052	0.0149, 0.0227	Rygge SAR	0.0000	0.0018, 0.0047	0.000
BMV > 12 yoa	SUH HEMS	0.0182	0.0143, 0.0220	OUH HEMS	0.0047	0.0036, 0.0058	0.000
	SUH HEMS	0.0182	0.0143, 0.0220	Rygge SAR	0.0017	0.0007, 0.0027	0.000
Invasive ventilator < 1 yoa	OUH HEMS	0.0015	0.0008, 0.0022	SUH HEMS	0.0000	0.0000, 0.0000	0.024
	OUH HEMS	0.0015	0.0008, 0.0022	Rygge SAR	0.0000	0.0000, 0.0000	0.023
Invasive ventilator 1–12 yoa	OUH HEMS	0.0015	0.0009, 0.0022	SUH HEMS	0.0000	0.0000, 0.0000	0.008
	OUH HEMS	0.0015	0.0009, 0.0022	Rygge SAR	0.0000	0.0000, 0.0000	0.007
Invasive ventilator > 12 yoa	OUH HEMS	0.0213	0.0190, 0.0237	SUH HEMS	0.0064	0.0043, 0.0086	0.000
	OUH HEMS	0.0213	0.0190, 0.0237	Rygge SAR	0.0012	0.0003, 0.0022	0.000
PVC 1–12 yoa	OUH HEMS	0.0024	0.0016, 0.0032	Rygge SAR	0.0003	−0.0001, 0.0007	0.007
PVC > 12 yoa	OUH HEMS	0.0153	0.0130, 0.0176	Rygge SAR	0.0052	0.0032, 0.0071	0.000
	SUH HEMS	0.0279	0.0226, 0.0332	OUH HEMS	0.0153	0.0130, 0.0176	0.000
	SUH HEMS	0.0279	0.0226, 0.0332	Rygge SAR	0.0052	0.0032, 0.0071	0.000
IO > 12 yoa	SUH HEMS	0.0029	0.0014, 0.0044	OUH HEMS	0.0008	0.0004, 0.0013	0.001
	SUH HEMS	0.0029	0.0014, 0.0044	Rygge SAR	0.0003	−0.0001, 0.0007	0.000
CVC insertion	OUH HEMS	0.0022	0.0014, 0.0029	SUH HEMS	0.0003	−0.0003, 0.0009	0.005
	OUH HEMS	0.0022	0.0014, 0.0029	Rygge SAR	0.0000	0.0000, 0.0000	0.001
Arterial line insertion	OUH HEMS	0.0053	0.0042, 0.0065	SUH HEMS	0.0009	0.0001, 0.0018	0.000
	OUH HEMS	0.0053	0.0042, 0.0065	Rygge SAR	0.0005	−0.0001, 0.0010	0.000
ACLS > 12 yoa	OUH HEMS	0.0084	0.0070, 0.0099	Rygge SAR	0.0031	0.0017, 0.0045	0.001
	SUH HEMS	0.0158	0.0124, 0.0192	OUH HEMS	0.0084	0.0070, 0.0099	0.000
	SUH HEMS	0.0158	0.0124, 0.0192	Rygge SAR	0.0031	0.0017, 0.0045	0.000
Fracture repositioned	SUH HEMS	0.0027	0.0012, 0.0041	Rygge SAR	0.0005	−0.0002, 0.0011	0.011
Dislocated joint repositioned	SUH HEMS	0.0008	0.0000, 0.0016	OUH HEMS	0.0001	−0.0001, 0.0002	0.004
	SUH HEMS	0.0008	0.0000, 0.0016	Rygge SAR	0.0000	0.0000, 0.0000	0.014
Gastric tube insertion	OUH HEMS	0.0028	0.0019, 0.0037	Rygge SAR	0.0003	−0.0001, 0.0007	0.007
	SUH HEMS	0.0031	0.0016, 0.0047	Rygge SAR	0.0003	−0.0001, 0.0007	0.014
Incubator with ventilator support	OUH HEMS	0.0023	0.0012, 0.0033	SUH HEMS	0.0000	0.0000, 0.0000	0.014
	OUH HEMS	0.0023	0.0012, 0.0033	Rygge SAR	0.0000	0.0000, 0.0000	0.015
Incubator with CPAP	OUH HEMS	0.0016	0.0008, 0.0024	SUH HEMS	0.0000	0.0000, 0.0000	0.022
	OUH HEMS	0.0016	0.0008, 0.0024	Rygge SAR	0.0000	0.0000, 0.0000	0.021
Incubator without ventilator	OUH HEMS	0.0011	0.0006, 0.0016	SUH HEMS	0.0002	−0.0002, 0.0005	0.039
	OUH HEMS	0.0011	0.0006, 0.0016	Rygge SAR	0.0000	0.0000, 0.0000	0.012
Antiarrythmic therapy	OUH HEMS	0.0016	0.0010, 0.0021	Rygge SAR	0.0003	−0.0001, 0.0007	0.035
Vasopressor initiated	OUH HEMS	0.0036	0.0027, 0.0045	SUH HEMS	0.0011	0.0002, 0.0020	0.003
	OUH HEMS	0.0036	0.0027, 0.0045	Rygge SAR	0.0005	−0.0001, 0.0010	0.000
Vasopressor continued	OUH HEMS	0.0087	0.0068, 0.0105	SUH HEMS	0.0022	0.0009, 0.0035	0.000
	OUH HEMS	0.0087	0.0068, 0.0105	Rygge SAR	0.0000	0.0000, 0.0000	0.000
Anasthesia induction	OUH HEMS	0.0148	0.0128, 0.0168	SUH HEMS	0.0082	0.0054, 0.0109	0.001
	OUH HEMS	0.0148	0.0128, 0.0168	Rygge SAR	0.0015	0.0005, 0.0026	0.000
	SUH HEMS	0.0082	0.0054, 0.0109	Rygge SAR	0.0015	0.0005, 0.0026	0.006
Pain management	OUH HEMS	0.0052	0.0040, 0.0065	Rygge SAR	0.0019	0.0003, 0.0034	0.021

*CI* Confidence Interval, *ETI* Endotracheal Intubation, *OUH* Oslo University Hospital, *SUH* Stavanger University Hospital, *HEMS* Helicopter Emergency Medical Service, Rygge *SAR* Search and Rescue helicopter at Rygge, *BMV* Bag Mask Ventilation, *yoa* years of age, *PVC* Peripheral Venous Catheter, *CVC* Central Venous Catheter, *IO* Intraosseus access, *ACLS* Advanced Cardiac Life Support, *pat* patient, *CPAP* Continous Positive Airway Pressure

## Discussion

Our data show that there are differences in the number of times a given procedure is performed prehospital, both between individual anaesthesiologists and between HEMS bases. Certain procedures are performed often, whereas others are rarely performed. Although there is conflicting evidence to support the effect of many advanced procedures in prehospital medicine, some critical procedures have been shown to contribute to better patient outcome or improve survival in prehospital patients when provided by specially trained physicians [1]. Among these procedures are ETI with the use of drugs [1, 7], chest tube drainage [1] and ACLS with advanced drug therapy [1, 8]. Our data suggest, that physicians working full time at all the bases studied perform all these procedures at least once a year on adolescent or adult patients, with the exception of chest tube drainage at Rygge SAR.

The reasons for the differences observed between the bases are probably multifactorial. One of these factors is the mission profile. Rygge SAR is primarily a SAR resource. Although it is part of the national air ambulance system in Norway, the frequency of ambulance missions is low and critical procedures are rarely performed. At OUH HEMS, one of the helicopters is mainly used for inter-facility transports. Consequently the frequency of procedures related to critical care is high, as is the case with invasive ventilator treatment and the use of vasopressors.

Differences in the patient population may also explain some of the differences. SUH HEMS has a significantly higher share of ACLS in adults than the other bases. Consequently, they also have a higher share of ETI and BMV in adults. The explanation for this remains speculative, but might have to do with a close proximity to a high-density urban area (the city of Stavanger) where the rapid response vehicle is often used. OUH HEMS is also located close to a high-density urban area (the city of Oslo), but is rarely used there because the city has a separate physician-staffed ambulance. On the other hand, OUH HEMS has a higher frequency of trauma ETIs and anaesthesia induction that might be explained with a high population of trauma patients in the OUS HEMS system since it covers a larger area with a larger population and more densely populated areas than SUH HEMS.

Other differences are more difficult to explain. The differences in frequency of procedures like central venous catheter insertion and arterial line insertion cannot be attributed to differences in population. In this case we speculate that attitudes of individual physicians and local culture may play a role. Previous studies have shown that even physician-staffed EMS does not always adhere to treatment guidelines indicating that individual opinions and system culture play a role in treatment strategies [9, 10]. We also know from risk assessment studies of critical procedures that culture and attitudes play a role in how critical procedures are carried out [11, 12]. Standard operating procedures and guidelines may explain some of the variance in how and when procedures are performed, but as a study from the Netherlands shows, even with established guidelines in place clinicians do not always adhere [9]. A risk assessment study of prehospital ETI also introduces “protocol compliance” as a factor that influences how procedures are carried out [11]. Training is probably a key to even out differences in both attitudes and culture, but it remains to be defined how much training is needed.

As our data and other recent studies show, prehospital physicians perform prehospital ETIs one to two times a month in the field [13]. The high success rates of prehospital ETI by prehospital anaesthesiologists suggest that this is sufficient [10, 13, 14]. A confounding factor is however, that most of these physicians have a much higher exposure to emergency ETI in their in-hospital practice [13]. What the relatively rare exposure of one ETI per month alone means for success rates therefore remains unknown.

Some of the procedures recorded in our study are outside the field of anaesthesiology per se, but are still elements of prehospital medicine delivered by physicians. Typical examples of this are birth assistance and incubator transports. As our data show, the exposure to these interventions and procedures is very low. For birth assistance our data indicate that a HEMS physician in Norway will actively assist in a delivery once every 10 – 20 years. This is probably explained partially by the fact that emergency medical technician and paramedic staffed ground ambulances in Norway are more often called out to prehospital childbirths and that HEMS physicians are rarely involved. Also, some HEMS systems in Norway have the opportunity to bring a midwife along to the patient. Still, it raises the question; to what extent should HEMS physicians train delivery procedures? This is a valid question since it can be expected that HEMS physicians will be called to the complicated cases.

Incubator transports are in Norway undertaken by various team setups. Some bases, like SUH HEMS, do not perform incubator transports; instead an incubator team consisting of a paediatrician and a neonatal nurse using other means of transport performs them. At OUH HEMS however, the HEMS physicians perform these transports themselves. As our data show, a prehospital physician at OUH HEMS will annually perform 6–7 incubator transports. Whether this exposure is sufficient to remain or indeed become proficient in the care for incubator patients remains to be answered. It does however raise the question if HEMS physicians should have formalised training and clinical experience outside the field of anaesthesiology alone, e.g. regular simulation based training or clinical practice in a neonatal ward.

It is difficult to conclude on how the relatively low exposure to certain procedures can be countered to maintain proficiency. Intuitively clinical practice in a setting that provides high volume in critical procedures seems reasonable; e.g. regular duty in an anaesthesiology department. The differences identified between the HEMS bases does however indicate that training needs might be different for each system and that it must be tailored to the local needs. The high number of prehospital ACLS cases in SUH HEMS as compared to OUH HEMS could indicate the need for a different approach to retraining at the two sites. Correspondingly, the high caseload of patients on invasive ventilator at OUH HEMS questions the need for exposure to critical care medicine in both training and clinical work in-hospital. We also know that even clinical practice in hospital is no guarantee for an appropriate caseload in emergency cases and correspondingly critical care procedures. Other means to ensure this must therefore be found. Simulation based training has the potential to provide health care professionals with a tailored learning experience and a planned and controllable exposure to certain patient cases and procedures. There are several good examples of simulation being used in the training and retraining of medical personnel in prehospital medicine [15]. Several studies have shown that simulation training in itself improves patient care and -outcome [16–18]. We therefore think that a tailored clinical practice supported by simulation training, with the possibility of individual adaptation, would be the optimal method to ensure sufficient proficiency and quality in the delivery of care by physicians in prehospital critical care medicine.

### Limitations

This study was performed in a limited time frame of 12 months at three sites. The data therefore represent a sample and can only be used for generalisation with caution. The low reporting rate at SUH HEMS and Rygge SAR also questions the validity of the data from these sites as compared to the high reporting rate from OUH HEMS. Still, we believe that with a reporting rate at almost 70 % the data from SUH HEMS and Rygge SAR can be trusted to be fairly representative.

The use of self-reported data has limitations and allows for reporting bias. However, since the data collection was made anonymously there is little reason for the individual physician to over- or under report his or her own activity. Accidental underreporting by unintended omission or oversight can be expected; the effect is however difficult to control and falls within the normal variation of this kind of data.

Since most of the physicians in our survey have duty in an anaesthesiology department as part of their rotation plan, this survey does not provide a correct impression of the total number of anaesthesiology related procedures performed in a year irrespective of pre- or in-hospital workplace. We therefore acknowledge that most of the physicians in this survey are probably proficient in anaesthesiology related procedures. This also supports our argument that clinical practice besides prehospital work is necessary and important. There still remains however, a relatively large amount of non-anaesthesiology related procedures were we believe our data is representative for the actual exposure frequency.

## Conclusion

HEMS anaesthesiologists in Norway perform various critical procedures at variable intervals and frequencies. Some skills and procedures known to increase survival are performed often, whereas others are performed more rarely. There are also differences between HEMS systems related to mission profile. HEMS systems should therefore tailor the off-duty clinical practice to the local mission profile and individual needs of the physicians.

## Additional file

Additional file 1: Table S1. The list of procedures that were recorded at the end of a shift or after no more than 24 hours when a shift lasted longer. A definition was offered for 7 procedures that needed more clarification.
