# Supplementary material for: A prospective survey of critical care procedures performed by physicians in helicopter emergency medical service: is clinical exposure enough to stay proficient?
Source: Scand J Trauma Resusc Emerg Med. 2015 Jun 11;23:45. doi: 10.1186/s13049-015-0128-9 (PMC4464230; doi:10.1186/s13049-015-0128-9)
Supplement: Additional file 1: Table S1. — The list of procedures that were recorded at the end of a shift or after no more than 24 hours when a shift lasted longer. A definition was offered for 7 procedures that needed more clarification. [file 13049_2015_128_MOESM1_ESM.docx]

| **Appendix 1.** The list of procedures that were recorded at the end of a shift or after no more than 24 hours when a shift lasted longer. A definition was offered for 7 procedures that needed more clarification | | |
| --- | --- | --- |
| **Procedures** | | |
| Endotracheal intubation,  0-1 years of age | Trauma | |
|  | Cardiac arrest | |
|  | Respiratory failure (non-traumatic) | |
|  | Other causes | |
| Endotracheal intubation,  1 - 12 years of age | Trauma | |
|  | Cardiac arrest | |
|  | Respiratory failure (non-traumatic) | |
|  | Other causes | |
| Endotracheal intubation,  > 12 years of age | Trauma | |
|  | Cardiac arrest | |
|  | Respiratory failure (non-traumatic) | |
|  | Other causes | |
| Supraglottic airway (i.e. LMA, LT) | 0-1 years of age | |
|  | 1-12 years of age | |
|  | > 12 years of age | |
| Coniotomy/ emergency tracheotomy | | |
| Bag mask ventilation | 0-1 years of age | |
|  | 1-12 years of age | |
|  | > 12 years of age | |
| Invasive ventilator | 0-1 years of age (excl. incubator) | |
|  | 1-12 years of age | |
|  | > 12 years of age | |
| Non-Invasiv respirator behandling | 0-1 years of age (excl. incubator) | |
|  | 1-12 years of age | |
|  | > 12 years of age | |
| Periferal venous access | 0-1 years of age | |
|  | 1-12 years of age | |
|  | > 12 years of age | |
| Intraosseus access | 0-1 years of age | |
|  | 1-12 years of age | |
|  | > 12 years of age | |
| Central venous catheter insertion | | |
| Aterial line insertion | | |
| Advanced cardiac life support | 0-1 years of age | |
|  | 1-12 years of age | |
|  | > 12 years of age | |
| External pacing | | |
| Needle chest decompression | | |
| Chest drainage (with or without chest drain insertion) | | |
| Other types of surgery, specify: | | |
| Reposition of dislocated fracture | | |
| Reposition of dislocated joint | | |
| Urethral catheter insertion | | |
| Gastric tube insertion | | |
| Incubator transport with ventilator support | | |
| Incubator transport with CPAP | | |
| Incubator transport without ventilator | | |
| Umbilical cord catheter insertion | | |
| Birth assistance | | |
| Inhalation therapy | | |
| Anti arrhythmic therapy | | |
| Local anaesthesia | | |
| Vasopressor initiated | | |
| Vasopressor continued | | |
| Anaesthesia induction | | |
| Advanced pain management | | |
|  | | |
| **Definitions** | | |
| Advanced cardiac life support | | Advanced cardiac life support with drugs and defubrillator according to current guidelines. |
| Incubator transport | | Transport carried out independently without neonatal team or paediatric support. |
| Inhalation therapy | | Treatment of e.g. obstructive condition with nebulized ipratropium bromide or salbutamol |
| Anti arrhythmic therapy | | Medical treatment of any type of tachycardic or bradycardic arrhythmia, excluding arrhythmias during cardiac arrest. |
| Local anaesthesia | | Any type of peripheral nerve block, e.g. femoral block. |
| Vasopressor | | Continuous vasopressor treatment, excluding bolus injections by e.g. cardiac arrest. |
| Advanced pain management | | Use of opioids or ketamine in analgetic doses by painful conditions or procedures, e.g. reposition of dislocated fracture, severe visceral pain. Includes the use of propofol for procedural sedation. |
